# To increase size or decrease density? Different *Microcystis* species has different choice to form blooms

**DOI:** 10.1038/srep37056

**Published:** 2016-11-14

**Authors:** Ming Li, Wei Zhu, Lili Guo, Jing Hu, Huaimin Chen, Man Xiao

**Affiliations:** 1 College of Resources and Environment, Northwest A & F University, Yangling 712100, PR China; 2 College of Environment, Hohai University, No. 1 Xikang Road, Nanjing 210098, PR China

## Abstract

The buoyancy of *Microcystis* colonies is a principal factor determining blooms occurrence but the knowledge of seasonal variation in buoyancy is quite poor because of challenge in analysis method. In this study, a method based on the Stokes’ Law after researching on the effects of shapes on settling velocity of *Microcystis* colonies, whose gas vesicles were collapsed, to accurately measure density was established. The method was used in Lake Taihu. From January to May, mean density of *Microcystis* colonies decreased from 995 kg m^−3^ to 978 kg m^−3^ and then increased to 992 kg m^−3^ in December. The density of colonies in different *Microcystis* species was in the order *M. wesenbergii* > *M. aeruginosa* > *M. ichthyoblabe*. For all the *Microcystis* species, the density of colonies with gas vasicles increased significantly along with the increase of colony size. Our results suggested that the main driving factor of *Microcystis* blooms formation in Lake Taihu was low density for *M. ichthyoblabe* from May to July but was large colony size for *M. wesenbergii* and *M. aeruginosa* from August to October.

*Microcystis* blooms is one of the most serious cyanobacterial blooms, which frequently occurs in freshwater ecosystems worldwide^1^,^2^. A mass of *Microcystis* biomass was nourished by the increasing nitrogen and phosphorus due to eutrophication^3^,^4^,^5^. However, the abrupt appearance of *Microcystis* blooms within a short period was due to the floating and aggregation of *Microcystis* colonies rather than its rapid growth^6^,^7^.

*Microcystis* could regulate buoyancy by collapse and synthesis of gas vesicles as well as accumulation and consumption of photosynthetic products such as polysaccharide and protein^8^. This function provided an essential factor for floatation and blooms formation of *Microcystis*. The buoyancy changes of *Microcystis* responding to varying environmental factors have been well studied and modeled^9^,^10^,^11^. However, most of these works just focused on diurnal variation in buoyancy. The knowledge of seasonal variation in buoyancy of *Microcystis* colonies is quite poor.

The analysis method is the major challenge which hinder to gather more information about buoyancy of *Microcystis* in lakes and reservoirs. So far, density gradient centrifugation method (DGC method) is the only way to directly measure density of *Microcystis* colonies^12^,^13^. However, this method could only be used to measure particles whose density is larger than water. This means this method could not analyze *Microcystis* colonies forming blooms with gas vesicles. The density of *Microcystis* colonies in lakes and reservoirs cannot be obtained yet.

Reynolds *et al.*^14^ described two methods to calculate colony density of *Microcystis*. The first method was by means of calculation from the relative volumes of mucilage, cells and gas vesicles. However, the relative volumes of mucilage, cells and gas vesicles were difficult to measure and the results were not reliable, either. The second method was calculating density from the floatation velocity base on the stokes’ law. Merely, the stokes’ law was the mostly used model to simulate the floatation and sedimentation. This model was based on two assumptions: i) all colonies were spheres; ii) the density of colonies with different size was same. Thereby, the floatation velocity was considered to be positively related to the square of colony size when the density of colonies were constant. However, Nakamura *et al.* reported that the regression between floatation velocity and colony size was not a quadratic relationship but linear in most cases^15^. Both of these results implied that the above two assumptions would be not appropriate.

Nakamura *et al.*^15^ suggested that the fractal dimension of *Microcystis* colonies was 2.5 and this value can be used in the stokes’ law to calculate density of *Microcystis*. However, the shape of *Microcystis* colonies used in their study was unclear. It was reported that the shape of *Microcystis* colonies were always irregular and the physiology of colonies with different size was also different^16^. Moreover, the shapes of the most common *Microcystis* species (*Microcystis aeruginosa*, *Microcystis wesenbergii* and *Microcystis ichthyoblabe*) differs significantly^17^. Therefore, it is necessary to assess the effects of shapes on floatation velocity of *Microcystis* colonies.

In addition, a lot of models have been established to simulate blooms formation based on the stokes’ law and buoyancy changes^18^,^19^,^20^. The density of *Microcystis* colonies in different shapes and size was assumed to be similar. However, this assumption has never been tested.

The aim of this study was to i) establish a method based on the stokes’ law after researching on the effects of shapes on floatation velocity of *Microcystis* colonies to accurately measure density of *Microcystis* colonies; ii) investigate seasonal variation in buoyancy of *Microcystis* colonies in fields and discuss its influencing factors; iii) analyze density of *Microcystis* colonies in different shapes and size. Lake Taihu, a shallow eutrophic lake in China, was selected as the study area. This is because Lake Taihu has been well studied and a series of data about environmental factors and biomass, colonial morphology and distribution of *Microcystis* could be obtained easily.

## Materials and Methods

The Stokes’ law described vertical migration velocity (v) of small and solid particle as:


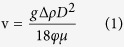


where μ is viscosity of water, g is acceleration of gravity, Δρ is effective density of the particle (Δρ = ρ_water_ − ρ_colony_ for a buoyant particle), D is diameter of particle, φ is shape coefficient, reflecting the influence of shape on migration velocity. The value of φ is 1 while the particle is small sphere. The parameters μ, g, ρ_water_ were constant and D could be measured directly via microscopes combined with image tools. The migaration velocity could be measured by many methods as well. Thus, if we can quantify φ, the density of *Microcystis* colony could be calculated as:


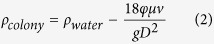


As per the DGC method could only be used to measure particles whose density is larger than water, *Microcystis* colonies without gas vesicles were used to quantify φ. *Microcystis* colonies collected from Lake Taihu was treated with a pressure of 0.65 MPa for 30 min using air compressor^9^. The density of the colonies without gas vesicles was measured by the DGC method. The distance divided by the time was calculated as v and this process was performed in a tube.

### *Microcystis* colonies collection

The sampling area was in Meiliang Bay of Lake Taihu where heavy *Microcystis* blooms occurred frequently in the recent years^21^. *Microcystis* colonies were directly collected from the surface blooms in Meiliang Bay of Lake Taihu in June, August and September 2012. Each sample was mainly constituted of *Microcystis ichthyoblabe*, *Microcystis wesenbergii* and *Microcystis aeruginosa*, respectively. The samples were equally divided into two groups: group A without any treatment; group B was treated with a pressure of 0.65 MPa for 30 minutes using air compressor to collapse all the gas vesicles.

### Analysis of floatation and settling velocity and colony size

Each group of sample was diluted by BG-11 culture medium until a single colony can be picked out by a pipette. Photomicrograph of the single colony was taken using an opticalC-5050 digital camera, and the colony size of *Microcystis* was directly analyzed using the UTHSCSA ImageTool program version3^22^. The length and width of *Microcystis* colonies were measured directly and the diameter of *Microcystis* colonies was calculated as diameter = (length × width)^1/2^.

The floatation velocity was analyzed via a glass tube closed at one end. The length of the tube was 70 cm and the inner diameter was 10 mm. The positions of the distance of 10 cm from both the ends of the tube were marked. The tube was filled with water and the open end was clogged by the thumb. Then, the open end was taken below the water surface in a water tank. Afterward, the tube was vertically fixed by a double-buret clrev. The colony was then re-pipetted and was injected into the bottom of the tube. It rise gradually and the floatation velocity keep in constant after floating up 10 cm. The time when the colony migrated between the two marks were recorded and the floatation velocity (mm s^−1^) was calculated as:


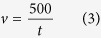


The settling velocity of *Microcystis* colonies without gas vesicles was measured by the same method but the tube was reversed.

### Density measurement of *Microcystis* colonies in group B

The DGC method was used to measure density of *Microcystis* colonies in group B. A density gradient column was prepared following the description of Miller and Gasek^12^. Mixtures of kerosene and carbon tetrachloride with different volume ration (75: 25 mL, 70: 30 mL, 65: 35 mL, 60: 40 mL, 55: 45 mL, 50: 50 mL; 45: 55 mL; 40: 60 mL; 35: 65 mL, respectively) were filled into a 1000 mL graduated cylinder successively from the bottom. After 24 hour’s standing, the density gradient column was demarcated by standard sucrose solutions at 20 °C.

A total of 15 mL Percoll layered liquid, 12 mL deionized water and 3 mL BG-11 medium was filled in a 50 mL centrifuge tube. Then, the tube was centrifuged at 4 °C with a speed of 25400× g for 2.0 h. After 1.5 hour’s standing at 20 °C, a *Microcystis* colony without gas vesicles, of which settling velocity has been measured, was gently transferred onto the surface of the mixture in the centrifuge tube. The tube was centrifuged at 20 °C with a speed of 3000 rpm for 20 min. Finally, two to three drops of the mixture in the same layer of the colony was injected in the density gradient column by a injection syringe. The average of the scale readings in the density gradient column were calculated as the density of *Microcystis* colony.

### Field investigation

Field investigation was carried out in the same area from January to December 2013. Sampling was carried out twice a month from July to October and then once a month for the rest of the period. Since the depths of sites were about 2 m, 500 mL of water was collected from the bottom to the water surface at 0.5 m intervals using a polymethyl methacrylate sampler^23^. It was kept at normal temperature and carried to the laboratory immediately for analysis of floatation velocity.

Water temperature was measured in the field using an electronic thermometer (Mettler SG7). Equal volumes of water collected at both the surface and a depth of 1.0 m below the surface were mixed in a 500 mL plastic bottle. It was kept refrigerated and carried to the laboratory for analysis of nutrient concentrations.

#### Analysis of nutrient concentrations

Half of the water samples were directly used to analyze total nitrogen (TN) and total phosphorus (TP) by colorimetry after digestion with K_2_S_2_O_8_+NaOH^24^. The other half of water samples for nutrient analysis were filtered through a 0.45 μm pore size membrane and the filtrate was used to analyze for total dissolved nitrogen (TDN) and total dissolved phosphorus (TDP) concentrations. The TDN and TDP concentrations were analyzed by colorimetry after digestion with K_2_S_2_O_8_+NaOH as well^24^.

## Results

### Floatation and settling velocity of different sized colonies

**Figure 1:**
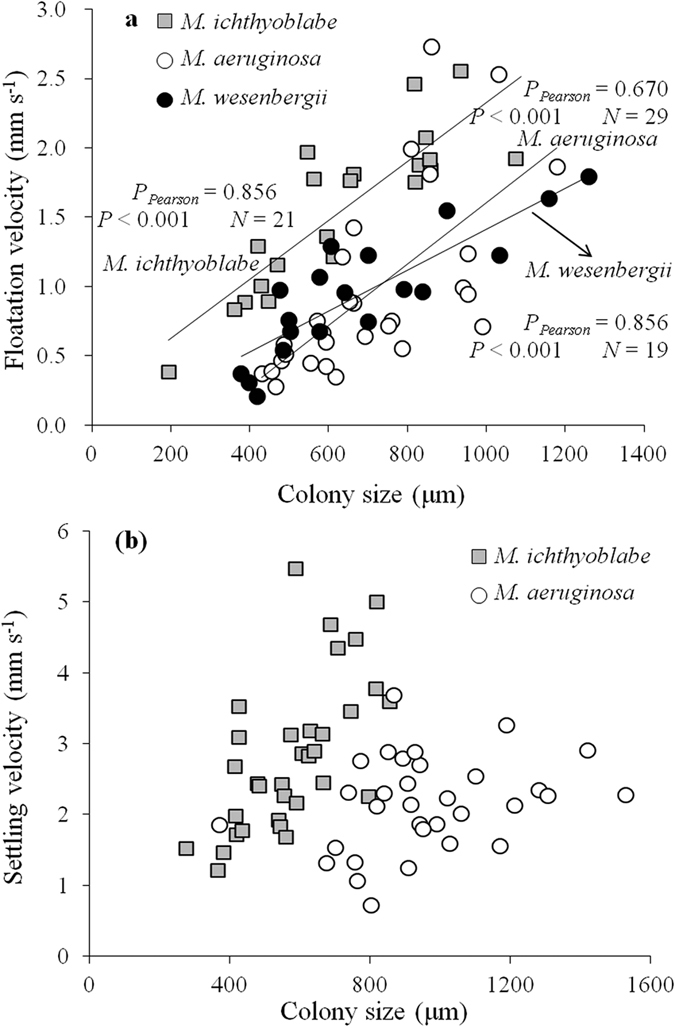


Figure 1 shows the floatation and settling velocity of different sized colonies of different *Microcystis* species. For all the *Microcystis* species, the floatation velocity of colonies with gas vasicles increased significantly along with the increase of colony size (Fig. 1a). The floatation velocity of *M. ichthyoblabe* colonies was much higher than colonies of both *M. aeruginosa* and *M. wesenbergii* if all the colonies were in the same size. The floatation velocity of *M. ichthyoblabe* colonies with diameters of 1000 μm reached 2.5 mm s^−1^.

However, the relationship between settling velocity and colony size of colonies without gas vasicles was not significant, especially for *M. aeruginosa (P*_*Pearson*_ = 0.106, *N* = 32). *M. wesenbergii* colonies had been broken while treated with high pressure and thus there was no data about *M. wesenbergii* in Fig. 1b.

### Shapes of *Microcystis* colonies

**Figure 2:**
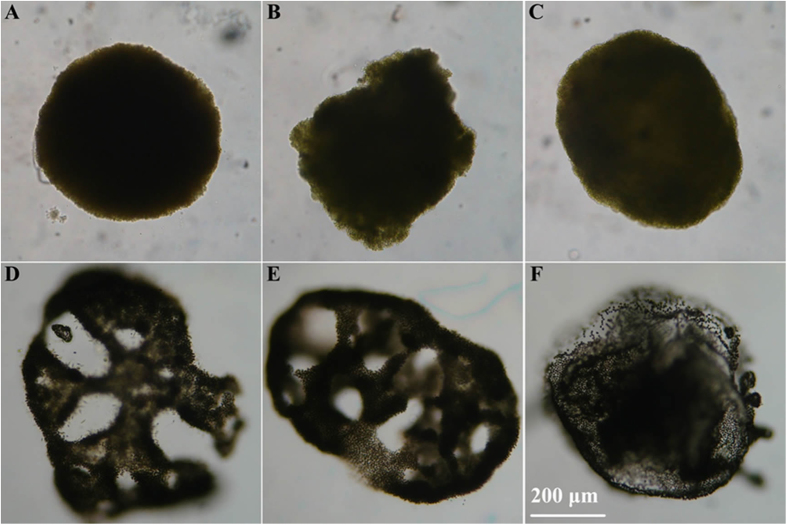


Colonies of *M. ichthyoblabe*, *M. aeruginosa* and *M. wesenbergii* were devided into a total of 6 types according to their shapes (Fig. 2). Type A is sphere *M. ichthyoblabe*; Type B is irregular *M. ichthyoblabe*; Type C is spheroid *M. ichthyoblabe*; Type D is irregular *M. aeruginosa*; Type E is spheroid *M. aeruginosa*; Type F is sphere *M. wesenbergii*. Irregular *M. wesenbergii* colonies were not involved in because it is difficult to be described. Futhermore, most *M. wesenbergii* colonies collected in the current study were sphere.

### Calculation of shape coefficient

The shape coefficient was considered be related only to shapes. In the current study, this coefficient was regressed by the diameter of *Microcystis* colonies. However, it did not mean that diameter of *Microcystis* colonies influence the shape coefficient. The underlying reason is that the diameter reflected to shapes of *Microcystis* colonies because the diameter was just quantified by the projection of three-dimensional *Microcystis* colonies.

Table 1 shows the regression results of the shape coefficient relating to diameter of *Microcystis* colonies. The shape coefficient of type A was constant (1.343 ± 0.019). Because *M. wesenbergii* colonies had been broken while treated with high pressure, no regression was performed to type F. Nevertherless, the value of type F was considered to be similar to type A because both of type A and F were sphere. For type B to E, the shape coefficient was significantly related to D^2^.

### Validation of the method

**Figure 3:**
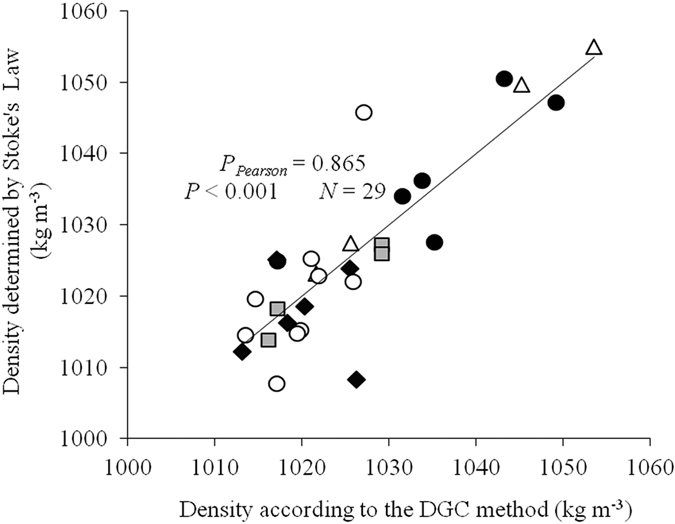


The relationship between the density calculated by our method and the density measured by the DGC method is shown in Fig. 3. The density calculated by our method was very close to the density measured by the DGC method.

### Density of *Microcystis* colonies relating to colony size

**Figure 4:**
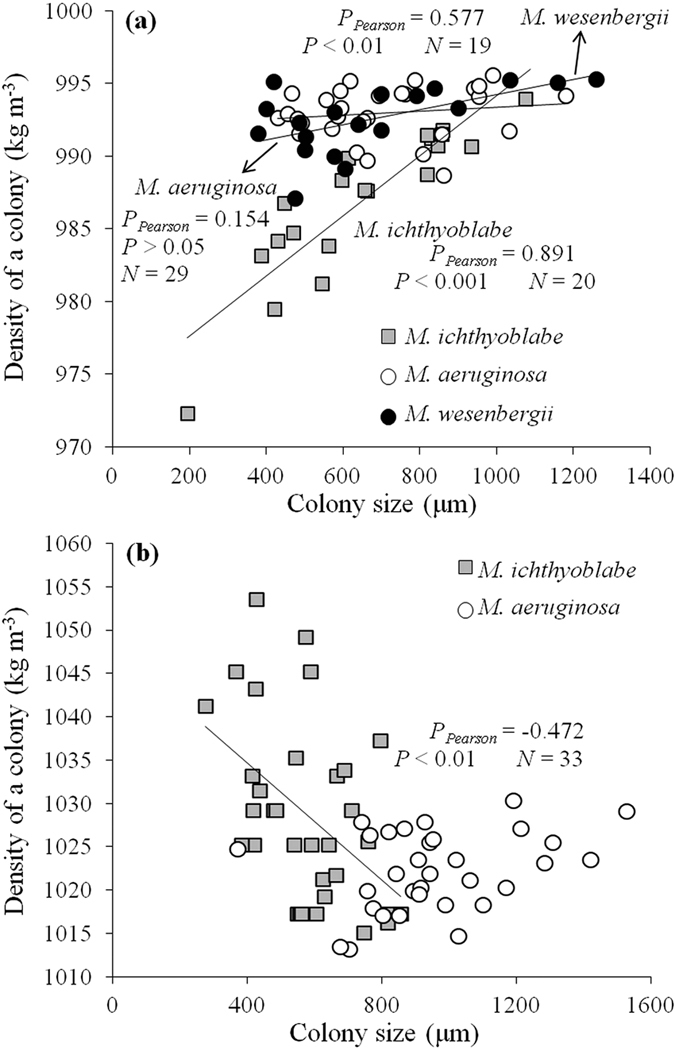


For all the *Microcystis* species, the density of colonies with gas vasicles increased significantly along with the increase of colony size except for *M. aeruginosa* (Fig. 4a). The density of *M. wesenbergii* and *M. aeruginosa* was in the range of 990 to 995 kg m^−3^ generally. However, the density of *M. ichthyoblabe* was much lower and the value was below 975 kg m^−3^ while the diameter was smaller than 200 μm.

The density of *M. aeruginosa* was in the range of 1015 to 1035 kg m^−3^ while their gas vesicles were collapsed by high pressure (Fig. 4b). However, the density incresed along with the decrease of colony size for *M. ichthyoblabe* colonies without gas vesicles. The maximum value was 1055 kg m^−3^.

### Seasonal variation in environmental factors

**Figure 5:**
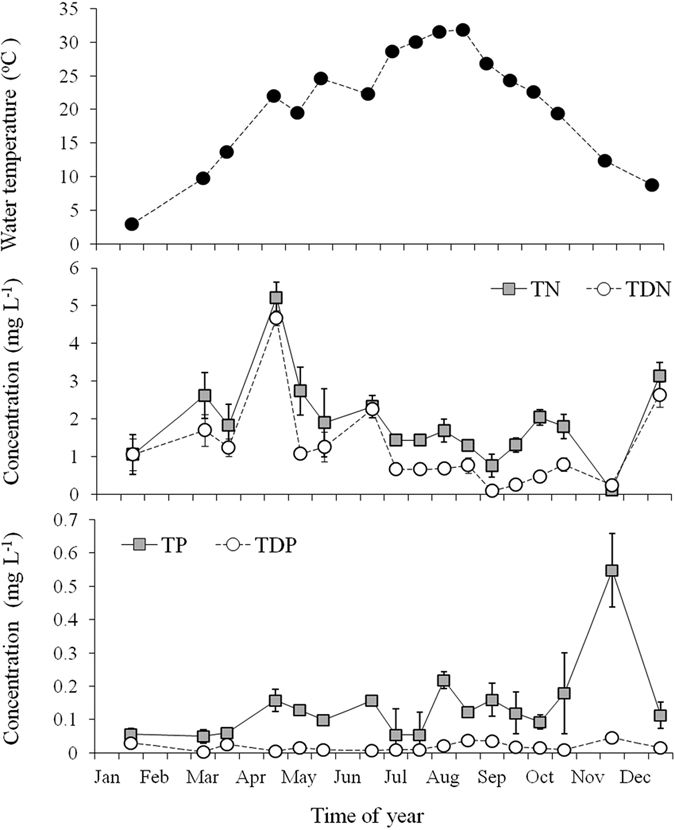


Water temperature and nutrient concentrations during the study period was illustrated in Fig. 5. Water temperature increased from 3.0 °C in January to 31.9 °C in August, and then decreased to 8.8 °C in December. The concentrations of TN and TDN were below than 2 mg L^−1^ at most of the time. The maximum concentations of TN and TDN were 5.2 mg L^−1^ and 4.7 mg L^−1^, respectively, both appeared in April. TP was smaller than 0.22 mg L^−1^ except for that in Novermber when the concentration was 0.55 mg L^−1^. The concentrations of TDP were always lower than 0.02 mg L^−1^.

### Seasonal variation in density of *Microcystis* colonies at varying depths

**Figure 6:**
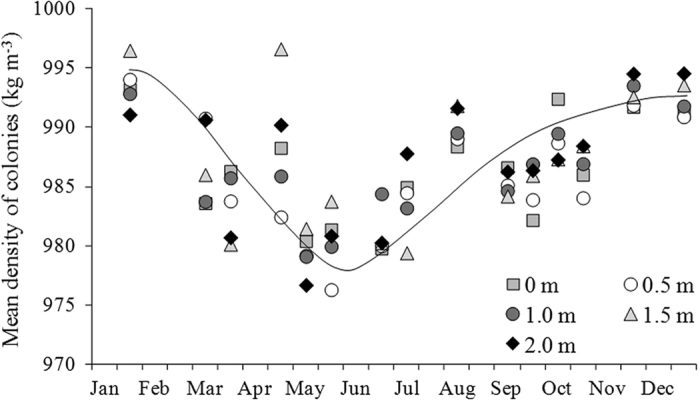


From January to May, mean density of *Microcystis* colonies decreased from 995 kg m^−3^ to 978 kg m^−3^ and then increased to 992 kg m^−3^ in December (Fig. 6). The density of colonies at varying depths were different but spatial distribution regularities are indistinct. The density of colonies at the water surface was always not the lowest in the whole water column. The maxmum and minimum density measured in the current study was 998.9 kg m^−3^ in January and 960.2 kg m^−3^ in May.

### Density of colonies in different *Microcystis* species

**Figure 7:**
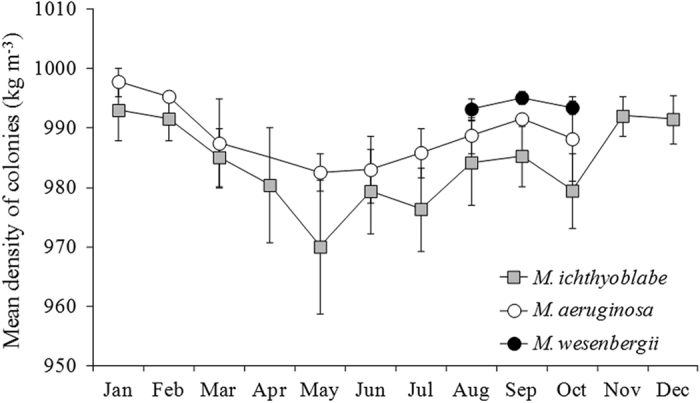


Density of both *M. ichthyoblabe* and *M. aeruginosa* colonies decreased form January to May and then inceased to Decmber (Fig. 7). The density of *M. wesenbergii* were 993–995 kg m^−3^ from August to October when *M. wesenbergii* could be found easily. Overall, density of colonies in different *Microcystis* species was in the order *M. wesenbergii* > *M. aeruginosa* > *M. ichthyoblabe*.

## Discussion

### Assessment of the approach

This study established a new systematic approach based on the stokes’ law for analysis buoyant density of *Microcystis* colonies. The floatation velocity, shapes and diameter of *Microcystis* colonies should be measured to calculate density. All these indicators were easily analyzed and the required equipments were just microscopes, cameras, tubes and a stopwatch. This easy-to-use approach is better than the method described by Reynolds *et al.* in which the relative volume of mucilage and gas volume should be quantified^14^. This is because that quantification of relative volume of mucilage and gas vesicles was extremely difficult and transmission electron microscope was required. Some researchers used the capillary compression tube to analyze the volume of gas vesicles but it was still difficult to accurately measure the volume of a small colony^10^.

This approach also demonstrated calculation of shape coefficient φ of *Microcystis* colonies based on the widely accepted stokes’ law. Three indicators including diameter, density and floatation velocity of colonies were measured to calibrate φ. The diameter was measured based on the projection of *Microcystis* colonies which will not reflect the real diameter of a colony. However, this gap was filled by shape coefficient because it is defined as a function of diameter in the current study. Similar relationship between shape coefficient and colony diameter was reported by Padisák *et al.*^25^. The density of *Microcystis* colonies without gas vesicles was measured by the DGC method. It is reported that the measuring error was ±0.001 kg m^−3^ 26. Thus, the value of density used in the calibration was credible. The measurement of floatation velocity of colonies was easy and the results would be dependable if the experiment was carried out under a quiet homothermal condition. The relationship between colony size and floatation velocity in the current study was similar to that in the work of Nakamura *et al.* which proved that the velocity measured in the current study was credible^15^. Overall, our approach is a credible easy-to-use method to analyze buoyant density of *Microcystis* colonies.

Nakamura *et al.* suggested that the fractal dimension of *Microcystis* colonies can be used in the stokes’ law to calculate density of *Microcystis*^15^. They reported that this value was 2.5 for large colonies but was 3 for small ones. However, most colonies used in their study was lower than 500 μm which were much smaller than that found in Lake Taihu^21^ and some other water systems^27^. The fractal dimension of *Microcystis* colonies were calculated as 1.4 (data not shown) in the current study according to the method of Nakamura *et al.*^15^. It could be deduced that the fractal dimension of *Microcystis* colonies decreased along with the increase of colony size.

### Density response to shapes and size of *Microcystis* colony

Our results also showed that density increased along with the increase of *Microcystis* colony size. The density of a colony was dependent upon the relative volume of mucilage and gas vesicles^14^. Even though, the density of gas vesicles was lower than 210 kg m^−3^ 9, it was deduced that the relative volume of gas vesicles was not related to colony size according to the results of Nakamura *et al.*^15^. Thus, the relative volume of gas vesicles would not contribute to variation in density of *Microcystis* colonies with different size.

Reynolds and Jaworski reported that the number of cells (N) in a *Microcystis* colony could be calculated by formula 4^28^:


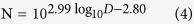


If the diameter of *Microcystis* cells was assigned as 7.2 μm, the relative volumes of mucilage of colonies with diameters of 100 μm and 500 μm were be calculated as 56.5% and 55.6%, respectively^15^. Moreover, our previous study proved that the intercellular space in a colony is high when colony size is high^29^. Both of these results proved that the relative volume of mucilage increased along with the increase of colony size. In addition, the density of mucilage was reported as 999.6 kg m^−3^ which was heavier than that of a buoyant *Microcystis* colony^14^. Therefore, there’s every reason to believe that density of *Microcystis* colonies increased along with the increase of colony size because of the increase of the relative volume of mucilage.

It was interesting that the density of colonies in different *Microcystis* species was in the order *M. wesenbergii* > *M. aeruginosa* > *M. ichthyoblabe*. Zhu *et al.* well discussed the distributianal difference between cells and the mucilaginous matrix composed of EPS in different morphological *Microcystis* colonies^30^. They reported that the extracellular polysaccharide (EPS) content of *Microcystis* species in the same size class was different with *M. aeruginosa* > *M. wesenbergii* > *Microcystis flos-aquae* (similar to *M. ichthyoblabe* in the current study). However, the intercellular space of *M. wesenbergii* was occupied by liquid similar to water, the density of which was similar to that of mucilage. Thus, it could be concluded that the contribution of mucilage and liquid occupied in the intercelluar space to the density of *Microcystis* colonies was in the order *M. wesenbergii* > *M. aeruginosa* > *M. ichthyoblabe*. In other words, the differences in density of *Microcystis* colonies in different shapes were owing to the differences in relative volume of mucilage.

### Seasonal variation in density of *Microcystis* colonies and its influencing factors

Reynolds and Rogers reported the percentages of colonies floating and sinking in Rostherne Mere^31^. Wang *et al.* also reported the floatation and settling velocity of *Microcystis* colonies in Lake Taihu^32^. However, this is the first report on seasonal variation in buoyant density of *Microcystis* colonies in lakes.

**Figure 8:**
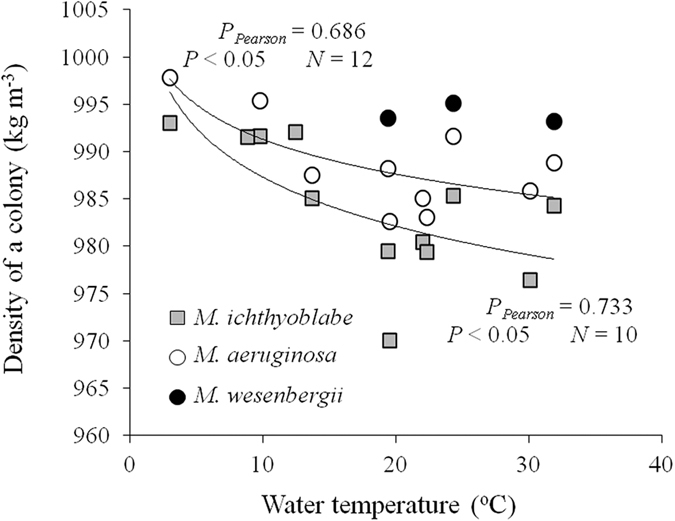


Colonies heavier than water were not recorded because the percentages of these heavy colonies were quite small in Lake Taihu^32^. The lowest density appeared in May and June. Similarly, Reynolds and Rogers also reported that the percentages of floating colonies in Rostherne Mere were largest (almost 100%) in June^31^. Therefore, the seasonal variation in buoyant density was that density decreased from January to May and then increased. No significant relationship between density and environmental factors were obtained except for temperature. It is obvious that the density decreased with increasing water temperature (Fig. 8).

It was also reported that N-limitation (<0.14 mg L^−1^) decreased the relative volume of gas vesicles by dilution of gas vesicles but the concentration of P did not affect the volume of gas vesicles^10^,^33^,^34^. In the current study, the TDN concentrations were always larger than 0.24 mg L^−1^ except for that in September (0.09 mg L^−1^). However, the density of *Microcystis* colonies were lower than that in both August and October, revealing that the formation of gas vesicles of *Microcystis* colonies in Lake Taihu during the investigation were not limited by nutrient. Light was also reported as an important factor influencing buoyancy of *Microcystis*^11^. But the mechanism is that high light intensity promoted the increase of ballast and thus resulted in losses of buoyancy^35^,^36^. This means that this mechanism is effective in the dual variation of buoyancy. However, the largest number of colonies were buoyant in the current study which indicated that this mechanism is not valid in the seasonal variation of density.

Kromkamp *et al.* reported that *M. aeruginosa* remained buoyant at 20 and 28 °C but reduced buoyancy at 15 °C^37^. The increase of density was caused by an increase of ballast and a decrease of gas vesicles. Thomas and Walsby found that gas vesicles formed at 20 °C but not did at 8 °C^38^. That is, high temperature is in favor of increase of ballast and formation gas vesicles and vice versa. It could deduced that increase of gas vesicles was slower than that of ballast with increasing temperature. Thus, the rate of density decline with increasing temperature was gradually decreased (Fig. 8).

### Contributions of buoyancy and colony size on blooms formation

The floatation velocity of *Microcystis* colonies combining lake mixing is the main mechanisms of abrupt *Microcystis* blooms formation^30^. Both buoyancy and colony size contributed to floatation velocity which affected blooms formation of *Microcystis*. It was reported that colony size increased until August and then decreased but the inflection point of density was in May^21^. From May to July, *M. ichthyoblabe* blooms occurred frequently whose density always below 980 kg m^−3^. From August to October, the main bloom-forming species was *M. wesenbergii* and *M. aeruginosa* of which the density was always larger than 990 kg m^−3^. Thus, it could deduced that the main driving factor of *Microcystis* blooms formation in Lake Taihu was low density from May to July but was large colony size from August to October.

This finding has guiding significance to control of *Microcystis* in Lake Taihu. The ultrasonic technique was well studied in the recent years which could collapse gas vesicles easily. This method is suited to use from May to July. The energy consumption of this method to collapse gas vesicle is low and it would be an environmentally friendly method if the strength was controlled to a certain extent. Moreover, the loss of buoyancy will also changes the dominated species in lakes^39^. The artificial mixture is a good approach to broke *Microcystis* colonies and this approach could be used from August to October. The combination of ultrasonic technique and artificial mixture would be an effective systematic method to control *Microcystis* blooms in temperate lakes and reservoirs.

## Additional Information

**How to cite this article**: Li, M. *et al.* To increase size or decrease density? Different *Microcystis* species has different choice to form blooms. *Sci. Rep.* **6**, 37056; doi: 10.1038/srep37056 (2016).

**Publisher’s note:** Springer Nature remains neutral with regard to jurisdictional claims in published maps and institutional affiliations.

## Figures and Tables

**Table 1:** The regression results of the shape coefficient relating to diameter of *Microcystis* colonies.

Type	Shape coefficient	D (μm)	R_Pearson_	P	N
A	φ = 1.343 ± 0.019	418~819			6
B	φ = 7 × 10^−6^ D^2^ + 0.192	276~797	0.953	<0.001	8
C	φ = 1 × 10^−6^ D^2^ + 1.281	384~856	0.928	<0.05	5
D	φ = 5 × 10^−6^ D^2^ − 0.285	773~1420	0.961	<0.001	8
E	φ = 7 × 10^−6^ D^2^ − 0.374	371~1529	0.989	<0.001	9
F	Not be analyzed (similar to A)				
